# Optimal EPO dosing in hemodialysis patients using a non-linear model predictive control approach

**DOI:** 10.1007/s00285-019-01429-1

**Published:** 2019-10-19

**Authors:** S. Rogg, D. H. Fuertinger, S. Volkwein, F. Kappel, P. Kotanko

**Affiliations:** 1 Fresenius Medical Care Deutschland GmbH, Bad Homburg, Germany; 2 Department for Mathematics and Statistics, University of Konstanz, Konstanz, Germany; 3 Institute for Mathematics and Scientific Computing, Karl-Franzens University of Graz, Graz, Austria; 4 Renal Research Institute, New York, NY USA; 5 Icahn School of Medicine at Mount Sinai, New York, NY USA

**Keywords:** Optimal control of hyperbolic equations, Model predictive control, PDE-constrained optimization, Quasi-Newton methods, Anemia, Erythropoietin, 35F45, 49J24, 49K20, 65K10, 90C30

## Abstract

Anemia management with erythropoiesis stimulating agents is a challenging task in hemodialysis patients since their response to treatment varies highly. In general, it is difficult to achieve and maintain the predefined hemoglobin (Hgb) target levels in clinical practice. The aim of this study is to develop a fully personalizable controller scheme to stabilize Hgb levels within a narrow target window while keeping drug doses low to mitigate side effects. First in-silico results of this framework are presented in this paper. Based on a model of erythropoiesis we formulate a non-linear model predictive control (NMPC) algorithm for the individualized optimization of epoetin alfa (EPO) doses. Previous to this work, model parameters were estimated for individual patients using clinical data. The optimal control problem is formulated for a continuous drug administration. This is currently a hypothetical form of drug administration for EPO as it would require a programmable EPO pump similar to insulin pumps used to treat patients with diabetes mellitus. In each step of the NMPC method the open-loop problem is solved with a projected quasi-Newton method. The controller is successfully tested in-silico on several patient parameter sets. An appropriate control is feasible in the tested patients under the assumption that the controlled quantity is measured regularly and that continuous EPO administration is adjusted on a daily, weekly or monthly basis. Further, the controller satisfactorily handles the following challenging problems in simulations: bleedings, missed administrations and dosing errors.

## Introduction

According to the 2018 United States Renal Data System annual data report (USRDS 2018), approximately 2.5 million patients were treated world-wide for end-stage renal disease in 2016. In nearly all reporting countries hemodialysis (HD) was the predominant form of dialysis therapy. On December 31, 2016, there were 726,331 prevalent cases of end-stage kidney disease in the U.S., of which 63.1% were receiving HD. Due to reduced erythropoietin production in the kidneys almost all HD patients suffer from a chronically decreased number of circulating red blood cells (RBCs) and associated low hemoglobin (Hgb) levels. This condition is called anemia. Untreated anemia is associated with poor quality of life and increased morbidity and mortality. Therefore, physicians aim for a partial correction of anemia with erythropoiesis stimulating agents (ESA). In this paper we consider the treatment with epoetin alfa (EPO), a human recombinant erythropoietin produced in cell culture.

Currently, the recommended Hgb target range for anemia management is 10–12 g/dl (see Mactier et al. 2011). Albeit, the KDIGO (Kidney Disease Improving Global Outcomes) Work Group, which provides clinical practice guidelines for anemia in chronic kidney disease and guidance on diagnosis, evaluation, management and treatment of HD patients, recommends not to exceed the limit of 11.5 g/dl in general but suggests to individualize therapy for patients whose quality of life may be improved at Hgb levels above 11.5 g/dl. Usually, dialysis facilities use dosing protocols that work as follows: A starting dose gets specified and based on the resulting Hgb change and depending on whether the patient is within the Hgb target range the ESA dose gets adjusted. This one-size-fits-all approach results in about 65% of patients achieving the set Hgb target. The Dialysis Outcomes and Practice Patterns Study (DOPPS) Practice Monitor reports that in the US, since December 2014, the percentage of patients with Hgb above 12 g/dl is 14–15% while 18–20% of patients have a Hgb below 10 g/dl. Moreover, Hgb variability and cycling are well known to occur in HD patients treated with ESA (Berns et al. 2003; Fishbane and Berns 2005, 2007). According to Yang et al. (2007) a greater Hgb variability is independently associated with higher mortality. The challenge in anemia treatment is the patients’ difference in long-term Hgb response to ESA. The drug concentration in plasma influences the maturation, proliferation and apoptosis of cells in the erythroid lineage. However, these cells remain about two weeks unobservable in the bone marrow before being released into the bloodstream. Consequently, it is difficult to anticipate the resulting delayed effect of the drug administration.

The mathematical model of erythropoiesis presented in Fuertinger et al. (2013) predicts patients’ erythropoietic response. We utilize this model to design a model-based feedback controller. The successful use of control algorithms for drug dosing has been shown, for example, in Magni et al. (2007) and in Bequette (2013). Both works are about closed-loop insulin dosing (i.e., the artificial pancreas). The control strategy we use is called model predictive control (MPC), also known as moving or receding horizon control. In 2011, Brier and Gaweda have already published an MPC-based algorithm for improved anemia management that has been tested and validated in clinical studies. Unlike our approach their predictive model is based on the concept of artificial neural networks (see also Barbieri et al. 2016; Brier et al. 2010). One of the limitations of using an artificial neural network approach is the need for large training and validation data sets making it very difficult to fully tailor this approach to individual patients and to personalize target Hgb ranges for patients whose quality of life would improve from a Hgb level higher than 11.5 g/dl as suggested by the KDIGO work group.

The purpose of this work is to develop a controller scheme that is fully personalizable. Previous to the study, the used model was adapted to individual patients using clinical data on Hgb levels and EPO administration. The details of the parameter estimation procedure and its results were published in Fuertinger et al. (2018). Note that insufficient iron availability is not modelled explicitly. However, several bone marrow parameters of the erythropoiesis model that are influenced by iron availability were estimated on a per patient level. The presented feedback controller is tested on various patient parameter sets. Throughout this manuscript we assume that EPO is administered continuously. This is currently not done in clinical practice but could be achieved with an “EPO pump” similar to the programmable insulin pumps used to treat diabetes mellitus. The approach is chosen nonetheless since it yields a continuous control which provides the best situation for stabilizing a system. Having developed a functional controller scheme it can then be adapted to actual dosing schedules and the effect of reducing the dosing frequency on the achievable stability can be analyzed. The given model equations are coupled hyperbolic partial differential equations (PDEs) and the control variable enters these equations non-linearly. In the presence of a non-linear model MPC is referred to as non-linear MPC (NMPC). More details on MPC can be found, e.g., in the books by Grüne and Pannek (2011) and by Rawlings and Mayne (2009). The basic principle of MPC consists of repeatedly solving finite horizon open loop optimal control problems. In each step, an open loop problem is solved. Then, only the first component of the obtained optimal control is applied and the optimization horizon gets pushed. This allows to include measurements and to react to unforeseen disturbances or complications. Here, we assume to know the true model and that we are able to measure Hgb perfectly. We simulate (gastro-intestinal) bleedings which are a common complication in HD patients and consider a malfunction of the pump by simulating the complete failure to administer EPO for an entire day or by applying an incorrect dose. Further, we simulate different frequencies of rate change with which the pump is programmed, ranging from daily to only once a month.

The paper is organized as follows: In Sect. 2 we introduce the control variable and present the model equations. The numerical approximation of these so-called state equations is investigated in Sect. 3. Both the model and its numerical approximation are recalled from Fuertinger et al. (2013). In addition, we regularize the erythrocytes model equation to obtain differentiability required for numerically solving the optimal control problem utilizing first-order optimality conditions. In Sect. 4 we formulate the optimal control problem and the NMPC algorithm is described. In Sect. 5 we present our numerical results of the following in-silico experiments: bleedings, missed administrations or wrongly administered doses and the restriction of EPO administration rates to be constant over several weeks. We draw some conclusions in Sect. 6. Finally, all parameters used for simulations are presented in Appendix A.

## The model of erythropoiesis

**Fig. 1:**
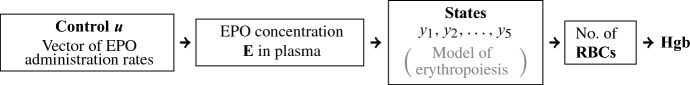
Schematics of the control structure

We start by describing the structure of the control process; compare Fig. 1. The *control* which is a vector of EPO administration rates can be altered to change a patient’s Hgb being the outcome. First, the EPO rates change the patient’s EPO concentration in plasma which affects the production of RBCs. The process of erythropoiesis is described by a mathematical model presented in Fuertinger et al. (2013). The different cell types during erythropoiesis are grouped into five population classes and the model allows to calculate their densities $y_1,y_2,\ldots ,y_5$. From a control perspective these are our *states*. Given the density $y_5$ of erythrocytes we can calculate their number and finally the Hgb.

In the following we explain the named components step by step. Note that the lemmas of this section will allow to utilize first-order optimality conditions for solving the optimal control problem numerically.

### The dosing of EPO as the control variable

We first write the time-varying EPO concentration in plasma as a function of EPO administration rates. Let us consider the time interval [0, *T*] with large final time $T\gg 1$. We assume that EPO can be applied continuously, with a constant administration rate per day or per multiple days. The EPO rates are given in U/day, where U stands for units. The EPO concentration *E*(*t*), $t\in [0,T]$, in plasma is separated into a constant summand $E^\mathrm {end}>0$ modeling the patient’s remaining endogenous erythropoietin level and a time-dependent summand $E^\mathrm {ex}(t)$ resulting from the administered EPO:

1 $$\begin{aligned} E(t) =E^\mathrm {end}+E^\mathrm {ex}(t)\quad \text {for }t\in [0,T]. \end{aligned}$$

Note that the endogenous EPO production $$E^\mathrm {end}$$ is estimated for each patient individually when adapting the model to patient data. For the number $$n_u\in {\mathbb {N}}$$ let the days $$\{t_u^j\}_{j=1}^{n_u+1}$$ with a constant EPO rate in $$[t_u^j,t_u^{j+1})$$, $$j=1,\ldots ,n_u$$, be given as

2 $$\begin{aligned} 0\le t_u^1<\cdots <t_u^{n_u+1}\le T. \end{aligned}$$

We introduce the associated finite-dimensional control space ${\mathscr {U}}={\mathbb {R}}^{n_u}$. Only non-negative EPO rates with a given upper positive limit $u_\mathrm {max}\in {\mathbb {R}}$ can be applied. Therefore, we are considering the (convex and compact) admissible set

$$\begin{aligned} {{\mathscr {U}}_\mathsf {ad}}=\big \{{{\varvec{u}}}=(u_j)_{1\le j\le n_u}\in {\mathscr {U}}\,\big |\,0\le {{\varvec{u}}}\le u_\mathrm {max}\big \}, \end{aligned}$$

where ‘$\le$’ is interpreted componentwise. Throughout, vectors are denoted by boldface letters. Let ${{\varvec{u}}}\in {{\mathscr {U}}_\mathsf {ad}}$ be given. Then, the time- and control-dependent summand $E^\mathrm {ex}=E^\mathrm {ex}(\cdot ;{{\varvec{u}}})$ satisfies the following initial value problem (cf. Fuertinger et al. 2013, equation (12)):

3 $$\begin{aligned} \begin{aligned} \frac{\mathrm d}{\mathrm d t}E^\mathrm {ex}(t;{{\varvec{u}}})&=\frac{1}{c_\mathsf {tbv}}\left( \sum _{j=1}^{n_u} u_j\chi _j(t)\right) -\lambda E^\mathrm {ex}(t;{{\varvec{u}}})\quad \text {for }t\in (0,T],\\ E^\mathrm {ex}(0;{{\varvec{u}}})&=E^\mathrm {ex}_\circ , \end{aligned} \end{aligned}$$

where

$$\begin{aligned} \chi _j=\chi _{[t_u^j,t_u^{j+1})},\quad j=1,\ldots ,n_u, \end{aligned}$$

are characteristic functions of the intervals $$[t_u^j,t_u^{j+1})$$, $$c_\mathsf {tbv}>0$$ stands for the total blood volume, $$E^\mathrm {ex}_\circ $$ is a non-negative initial condition for the exogenous EPO level and $$\lambda = \log (2)/T_{1/2}>0$$ is the EPO degradation rate with half-life time $$T_{1/2}$$. Thus, for $$t\in [t_u^j,t_u^{j+1})$$, $$j=1,\ldots ,n_u$$, the EPO concentration is given by

4 $$\begin{aligned} \begin{aligned} E(t;{{\varvec{u}}})&= E^\mathrm {end}+ e^{-\lambda t}E^\mathrm {ex}_\circ \\&\quad +\frac{e^{-\lambda t} }{c_\mathsf {tbv}\lambda }\left( u_j\big (e^{\lambda t} - e^{\lambda t_u^j}\big )+\sum _{i=1}^{j-1} u_i\big (e^{\lambda t_u^{i+1}} - e^{\lambda t_u^i}\big )\right) . \end{aligned} \end{aligned}$$

It follows from (4) that ${{\varvec{u}}}\mapsto E(\cdot ;{{\varvec{u}}})$ is a function mapping from the admissible set ${{\mathscr {U}}_\mathsf {ad}}\subset {\mathscr {U}}$ into *C*([0, *T*]), where *C*([0, *T*]) is the space of all continuous functions from [0, *T*] to ${\mathbb {R}}$. From (4) and $\lambda >0$ we infer that

$$\begin{aligned} E(t;{{\varvec{u}}})\ge E^\mathrm {end}+e^{-\lambda T}E^\mathrm {ex}_\circ =:E_\mathrm {min}>0\quad \text {for }t\in [0,T]\text { and }{{\varvec{u}}}\in {{\mathscr {U}}_\mathsf {ad}}. \end{aligned}$$

Thus, we define the interval of possible EPO concentrations

$$\begin{aligned} {{\mathscr {E}}_\mathsf {ad}}=[E_\mathrm {min},\infty )=\big \{E\in {\mathbb {R}}\,\big |\,E\ge E_\mathrm {min}\big \} \end{aligned}$$

and observe that $E(t;{{\varvec{u}}})\in {{\mathscr {E}}_\mathsf {ad}}$ holds for all $t\in [0,T]$ and ${{\varvec{u}}}\in {{\mathscr {U}}_\mathsf {ad}}$.

#### Lemma 1

The mapping *E* introduced in (4), has the following properties.

1. For every ${{\varvec{u}}}\in {{\mathscr {U}}_\mathsf {ad}}$ the function $E(\cdot ;{{\varvec{u}}}):[0,T]\rightarrow {\mathbb {R}}$ is continuously differentiable.
2. The mapping $E(t;\cdot ):{{\mathscr {U}}_\mathsf {ad}}\rightarrow {\mathbb {R}}$ is twice continuously differentiable for any $t\in [0,T]$. Its gradient is given as
  $$\begin{aligned} \nabla _{{\varvec{u}}}E(t;{{\varvec{u}}})=\frac{e^{-\lambda t}}{c_\mathsf {tbv}\lambda }\left( \begin{array}{c} e^{\lambda t_2}-e^{\lambda t_1}\\ \vdots \\ e^{\lambda t_i}-e^{\lambda t_{i-1}}\\ e^{\lambda t} - e^{\lambda t_i}\\ 0\\ \vdots \\ 0 \end{array} \right) \in {\mathscr {U}} \end{aligned}$$
  at $t\in [t_u^j,t_u^{j+1})$, $j=1,\ldots ,n_u$, and ${{\varvec{u}}}\in {{\mathscr {U}}_\mathsf {ad}}$. Further, the hessian matrix $\nabla ^2_uE(t;{{\varvec{u}}})\in {\mathbb {R}}^{n_u\times n_u}$ is zero.

#### Proof

The claims follow directly from formula (4). Since $\nabla _{{\varvec{u}}}E(t;{{\varvec{u}}})$ is independent of ${{\varvec{u}}}$, the hessian $\nabla ^2_uE(t;{{\varvec{u}}})$ is zero. $\square$

### The PDE model of erythropoiesis

**Fig. 2:**
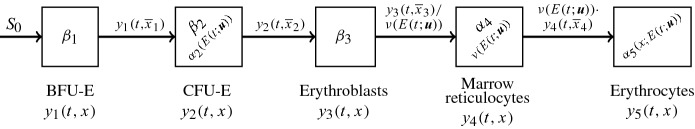
Schematic of the PDE model of erythropoiesis

A schematic of the model is shown in Fig. 2. For the underlying assumptions and more details we refer to Fuertinger (2012), Fuertinger et al. (2013). Stem cells commit to the erythroid lineage at a constant rate $S_0>0$. Once a stem cell has committed it passes the five shown cell classes/stages over time (if it does not die along the way): BFU-E (burst-forming unit erythroids), CFU-E (colony-forming unit erythroids), erythroblasts, marrow reticulocytes and erythrocytes (including blood reticulocytes). This means that there is a flux of cells from each population class to the subsequent one. For example, when a CFU-E cell has reached maximum age of that class it leaves the class and becomes an erythroblast with minimum maturity. The population densities depend on maturity and time. For each class an age-structured population model is given which describes the development of the respective class subject to a given EPO concentration in plasma. EPO has a direct effect on the rate of apoptosis of CFU-E cells ($\alpha _2$), the maturation velocity of marrow reticulocytes ($\nu$) and the mortality rate of erythrocytes ($\alpha _5$). For each class we are given an individual maturity interval $\varOmega _i=({{\underline{x}}}_i,{{\overline{x}}}_i)\subset {\mathbb {R}}$, $1 \le i \le 5$, in days. The interval boundaries are given by ${{\underline{x}}}_1=0$, ${{\overline{x}}}_1=3 = {{\underline{x}}}_2$, ${{\overline{x}}}_2 = 8 = {{\underline{x}}}_3$, ${{\overline{x}}}_3=13 = {{\underline{x}}}_4$, ${{\overline{x}}}_4=15.5$, ${{\underline{x}}}_5 =0$, whereas the RBC lifespan ${{\overline{x}}}_5$ is patient-dependent. Ma et al. (2017) have measured this shortened RBC lifespan in HD patients to be in the range of 37.7 to 115.8 days. Note that the first four cell classes are in bone marrow while the fifth class describes cells circulating in blood. That is why ${{\underline{x}}}_5$ is set to 0.

For conciseness , we write all five state equations in one form. Suppose that for given ${{\varvec{u}}}\in {{\mathscr {U}}_\mathsf {ad}}$ and $E^\mathrm {ex}_\circ \ge 0$ the EPO concentration $E=E(t;{{\varvec{u}}})$ is given as (4). Then, the equation reads 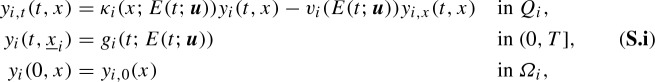 with the maturity interval $\varOmega _i=({{\underline{x}}}_i,{{\overline{x}}}_i)\subset {\mathbb {R}}$, the cylinder $Q_i=(0,T)\times \varOmega _i$ and the initial condition $y_{i,0}$. The solution $y_i(t,x)$ to (**S.i**) denotes the cell density of the respective cell population with maturity *x* at time *t*. The function $v_i$ describes the maturation velocity and $\kappa _i(\cdot )$ is of form $\beta _i - \alpha _i(\cdot )$, where $\beta _i > 0$ describes the profileration rate and $\alpha _i$ the rate of apoptosis. Actually, the function $\alpha _5$ and the sigmoid functions $\alpha _2$ and $\nu$ depend on the bounded (patient-dependent) parameter vector ${\varvec{\mu }}=(\mu _i)\in {\mathbb {R}}^{10}_+$ with ${\mathbb {R}}_+=\{s\in {\mathbb {R}}\,|\,s>0\}$. To simplify the notation we do not indicate dependencies on ${\varvec{\mu }}$. We refer to Appendix A, where all fixed and all individualized parameters, which we utilize in our numerical experiments, are listed. The individualized parameters are obtained via parameter estimation; see Fuertinger et al. (2018). The functions for the different classes read as follows:

5 $$\begin{aligned} \begin{aligned}&v_i(E)=\left\{ \begin{aligned}&\nu (E)&\text {if }i=4,\\&1&\text {otherwise}, \end{aligned} \right.&\kappa _i(x;E)=\left\{ \begin{aligned}&\beta _1&\text {if }i=1,\\&\beta _2-\alpha _2(E)&\text {if }i=2,\\&\beta _3&\text {if }i=3,\\&-\alpha _4&\text {if }i=4,\\&-\alpha _5(x;E)&\text {if }i=5, \end{aligned} \right. \\&y_{i,0}(x)=y_{i,0}(x)\text { for }i=1,\ldots ,5,&g_i(t;E)=\left\{ \begin{aligned}&S_0&\text {if }i=1,\\&y_1(t,{{\overline{x}}}_1)&\text {if }i=2,\\&y_2(t,{{\overline{x}}}_2)&\text {if }i=3,\\&y_3(t,{{\overline{x}}}_3)\big /\nu (E)&\text {if }i=4,\\&\nu (E)\,y_4(t,{{\overline{x}}}_4)&\text {if }i=5, \end{aligned} \right. \end{aligned} \end{aligned}$$

where, for $E\in {{\mathscr {E}}_\mathsf {ad}}$, the real-valued functions $\alpha _2$ and $\nu$ are given by

6 $$\begin{aligned} \alpha _2(E)=\frac{\mu _1}{1+e^{\mu _2E-\mu _3}}, \quad \nu (E)=\frac{\mu _4-\mu _5}{1+e^{-\mu _6E+\mu _7}}+\mu _5, \end{aligned}$$

and the function

$$\begin{aligned} \begin{aligned}&\alpha _5:\varOmega _5\times {{\mathscr {E}}_\mathsf {ad}}\rightarrow {\mathbb {R}},\\&\alpha _5(x;E)=\alpha _5^0+ \left\{ \begin{aligned}&\min \bigg (\frac{\mu _8}{E^{\mu _9}},\mu _{10}\bigg )&\text {if } x\in {\widehat{\varOmega }}_5,~E\in {{\mathscr {E}}_\mathsf {ad}}\text { with }E\le \tau _E,\\&0&\text {otherwise}, \end{aligned} \right. \end{aligned} \end{aligned}$$

stands for the erythrocytes mortality rate with an EPO threshold $\tau _E>0$ for neocytolysis. The (closed) non-empty interval ${\widehat{\varOmega }}_5 \subsetneq \varOmega _5$ denotes the age interval, where neocytolysis is possible.

#### Lemma 2

The mappings $\alpha _2,\nu :{{\mathscr {E}}_\mathsf {ad}}\rightarrow {\mathbb {R}}$ are continuously differentiable.

#### Proof

The claim follows directly from (6). $\square$

We denote the coupled system (**S.1**)–(**S.5**) by (**S**).

### Total RBC population

If the erythrocytes population density $y_5$ is known, the *total RBC population*$P=P(t)$, $t\in [0,T]$, is given as

7 $$\begin{aligned} P[{{\varvec{y}}}](t)=\int _{\varOmega _5}y_5(t,x)\,\mathrm {d}x\quad \text {for }t\in [0,T], \end{aligned}$$

where ${{\varvec{y}}}=(y_i)_{1\le i\le 5}$ solve the state system (**S**).

### Hgb concentration

Given a patient’s total blood volume $c_\mathsf {tbv}$ (in ml) together with the total number of RBCs *P*, the Hgb concentration (in g/dl) is calculated as

8 $$\begin{aligned} \mathrm {Hgb}\, = \frac{P \cdot \mathrm {MCH}}{c_\mathsf {tbv}\cdot 10^{10}}, \end{aligned}$$

where $\mathrm {MCH} = 29$ pg denotes the mean corpuscular hemoglobin.

### Regularization of the equation for the erythrocytes

In Sect. 4 we formally introduce the non-linear optimal control problem. In order to solve it numerically by utilizing first-order necessary optimality conditions we have to differentiate the state system (**S**) with respect to the state variable ${{\varvec{y}}}=(y_i)_{1\le i\le 5}$ and the control variable ${{\varvec{u}}}=(u_j)_{1\le j\le n_u}$. From Lemma 1 we already know that ${{\varvec{u}}}\mapsto E(t;{{\varvec{u}}})$ is continuously differentiable for every $t\in [0,T]$. Moreover, the mappings $\alpha _2$ and $\nu$ are continuously differentiable by Lemma 2. However, the mapping ${{\mathscr {E}}_\mathsf {ad}}\ni E\mapsto \alpha _5(x;E)$ is non-differentiable for every $x\in {\widehat{\varOmega }}_5$. Therefore, we have to regularize $\alpha _5$ in order to get smooth state equations.

For that reason we introduce the Heaviside function $H:{\mathbb {R}}\rightarrow {\mathbb {R}}$ defined as

$$\begin{aligned} H(s)=0\text { for }s\le 0\quad \text {and}\quad H(s)=1\text { for }s >0. \end{aligned}$$

Then, the mortality rate can equivalently be written as

9 $$\begin{aligned} \alpha _5(x;E)=\alpha _5^0+\chi _{{\widehat{\varOmega }}_5}(x)H(\tau _E-E)R(E),\quad \text {for }x\in \varOmega _5\text { and }E\in {{\mathscr {E}}_\mathsf {ad}}\end{aligned}$$

with

10 $$\begin{aligned} R(E)=\min \bigg (\frac{\mu _8}{E^{\mu _9}},\mu _{10}\bigg )\quad \text {for }E\in {{\mathscr {E}}_\mathsf {ad}}. \end{aligned}$$

For $\varepsilon >0$ we utilize the following regularized Heaviside function $H^\varepsilon :{\mathbb {R}}\rightarrow {\mathbb {R}}$

$$\begin{aligned} H^\varepsilon (s)=\left\{ \begin{aligned}&0&\text {if }s\le 0,\\&\frac{s^4}{\varepsilon ^6}\,\big (10s^2-24\varepsilon s+15\varepsilon ^2\big )&\text {for }s\in (0,\varepsilon ),\\&1&\text {if }s\ge \varepsilon , \end{aligned} \right. \end{aligned}$$

which is twice continuously differentiable. Notice that the function

$$\begin{aligned} F^\varepsilon (s,\tau )=(s-\tau )H^\varepsilon (\tau -s) + \tau ,\quad s,\tau \in {\mathbb {R}}\end{aligned}$$

is an approximation of $\min (s,\tau )$. Thus, the function *R* defined in (10) can be regularized as

$$\begin{aligned} R^\varepsilon (E) = F^\varepsilon \bigg (\frac{\mu _8}{E^{\mu _9} }, \mu _{10}\bigg )\quad \text {for }E\in {{\mathscr {E}}_\mathsf {ad}}\end{aligned}$$

which allows us to replace the non-smooth coefficient function $\alpha _5$ by the smooth (with respect to *E*) mapping

11 $$\begin{aligned} \alpha _5^\varepsilon (x;E)=\alpha _5^0+\chi _{{\widehat{\varOmega }}_5}(x)H^\varepsilon (\tau _E-E) R^\varepsilon (E)\quad \text {for }x\in \varOmega _5\text { and }E\in {{\mathscr {E}}_\mathsf {ad}}. \end{aligned}$$

#### Lemma 3

For every $x\in \varOmega _5$ the mapping $\alpha _5^\varepsilon (x;\cdot ):{{\mathscr {E}}_\mathsf {ad}}\rightarrow {\mathbb {R}}$ is continuously differentiable.

#### Proof

The claim follows directly from (11) because of $E_\mathrm {min}>0$. $\square$

In the sequel we replace $\alpha _5$ in (5) by $\alpha _5^\varepsilon$ and hence $\kappa _5$ by $\kappa _5^\varepsilon$ to account for the regularized fifth state equation which we denote by ($\mathbf {S.5}^\varepsilon$). Let (${\mathbf {S}}^\varepsilon$) be the state system (**S.1**)–(**S.4**) and ($\mathbf {S.5}^\varepsilon$).

## Numerical approximation of the state equations

The numerical solution of the age-structured population models is based on semigroup theory. We formulate the five state equations as abstract Cauchy problems which are then approximated by semigroups acting on finite dimensional subspaces. We have compared this discretization to an upwind finite difference scheme (cf. Strikwerda 2004). Due to computational speed while obtaining a similar approximation quality we have favored the semigroup based approach. We refer the reader to, e.g., Ito and Kappel (2002) and Kappel and Zhang (1993) for results on evolution operators and their approximation.

In the following we derive the discretization of the state equations by means of the general form (**S.i**) where we omit the dependency on *i* but replace $\kappa$ by $\kappa ^\varepsilon$.

### The state equations as abstract Cauchy problems

For $n \in {\mathbb {N}}$ we introduce the function

$$\begin{aligned} \delta _n(x)=\left\{ \begin{aligned}&-2n^2\bigg (x-{{\underline{x}}}-\frac{1}{n}\bigg )&\text {for }{{\underline{x}}}\le x\le {{\underline{x}}}+\frac{1}{n},\\&0&\text {for }{{\underline{x}}}+\frac{1}{n}< x\le {{\overline{x}}}, \end{aligned} \right. \end{aligned}$$

where $({{\underline{x}}},{{\overline{x}}}) = \varOmega$ denotes the maturity interval of the respective cell class. Hence, the sequence $\{\delta _n\}_{n\in {\mathbb {N}}}$ approximates the $\delta$-distribution. Let $y_n$, $n\in {\mathbb {N}}$, be a mild solution of the non-homogeneous Cauchy problem

12 $$\begin{aligned} \frac{\mathrm d}{\mathrm dt}\,y_n(t)={\mathcal {A}}^\varepsilon (E(t;{{\varvec{u}}}))y_n(t)+g(t;E(t;{{\varvec{u}}}))\delta _n\text { for }t\in (0,T],\quad y_n(0)=y_0 \end{aligned}$$

for given ${{\varvec{u}}}\in {{\mathscr {U}}_\mathsf {ad}}$, where the linear operator ${\mathcal {A}}^\varepsilon (E)$, $E\in {{\mathscr {E}}_\mathsf {ad}}$, is defined as

$$\begin{aligned}&\mathrm {dom}\,{\mathcal {A}}^\varepsilon (E)=\big \{\varphi \in L^2(\varOmega )\,\big |\,\varphi \text { is absolutely continuous on }{\overline{\varOmega }},\\&\quad \varphi ({{\underline{x}}})=0,v(E)\varphi '-\kappa ^\varepsilon (\cdot ;E)\varphi \in L^2(\varOmega )\big \},\\&\quad {\mathcal {A}}^\varepsilon (E)\varphi =-v(E)\varphi '+\kappa ^\varepsilon (\cdot ;E) \varphi \text { for }\varphi \in \mathrm {dom}\,{\mathcal {A}}^\varepsilon (E). \end{aligned}$$

It can be shown that $\lim _{n\rightarrow \infty }y_n(t)=y(t)$ in $L^2(\varOmega )$ holds for $t\in [0,T]$, where *y* is the solution to the corresponding state equation (**S.1**)–(**S.4**) or ($\mathbf {S.5}^\varepsilon$).

Since the range for the attribute is different for the cell populations considered, it is useful to normalize these attributes such that the range of the normalized attribute $\xi$ is [0, 1]. In order to achieve this we set $w={{\overline{x}}}-{{\underline{x}}}>0$ and define the mapping

$$\begin{aligned} h:[0,1]\rightarrow {\overline{\varOmega }}=[{{\underline{x}}},\overline{x}],\quad h(\xi )={{\underline{x}}}+w\xi \quad \text {for }\xi \in [0,1] \end{aligned}$$

with its inverse given by

$$\begin{aligned} h^{-1}:{\overline{\varOmega }}\rightarrow [0,1],\quad h^{-1}(x)=\frac{x-{{\underline{x}}}}{w}\quad \text {for }x\in {\overline{\varOmega }}. \end{aligned}$$

We denote by $L^2_w(0,1)$ the Hilbert space $L^2(0,1)$ endowed with the weighted inner product

$$\begin{aligned} {\langle {{\tilde{\varphi }}},{{\tilde{\phi }}}\rangle }_w=w\int _0^1{{\tilde{\varphi }}}(\xi ){{\tilde{\phi }}}(\xi )\,\mathrm d\xi =w\,{\langle {{\tilde{\varphi }}},{{\tilde{\phi }}}\rangle }_{L^2(0,1)} \quad \text {for }{{\tilde{\varphi }}},{{\tilde{\phi }}}\in L^2_w(0,1). \end{aligned}$$

The induced norm is $\Vert \cdot \Vert _w=w^{1/2} \Vert \cdot \Vert _{L^2(0,1)}$. The Hilbert spaces $L^2_{\omega }(0,1)$ and $L^2(\varOmega )$ are isomorphic with the isomorphism $\varXi :L^2(\varOmega )\rightarrow L^2_w(0,1)$ and its inverse $\varXi ^{-1}:L^2_w(0,1)\rightarrow L^2(\varOmega )$ given by

$$\begin{aligned} \varXi \varphi =\varphi \circ h\text { for }\varphi \in L^2(\varOmega ),\quad \varXi ^{-1}{{\tilde{\varphi }}}={{\tilde{\varphi }}}\circ h^{-1}\text { for }{{\tilde{\varphi }}}\in L^2_w(0,1). \end{aligned}$$

Applying the operator $\varXi$ to the sequence $\{\delta _n\}_{n\in {\mathbb {N}}}$ yields

$$\begin{aligned} {{\tilde{\delta }}}_n(\xi )=(\varXi \delta _n)(\xi )=\left\{ \begin{aligned}&-2n^2\bigg (w \xi -\frac{1}{n}\bigg )&\text {for }0\le w\xi \le \frac{1}{n},\\&0&\text {for }\frac{1}{n}< w\xi \le 1. \end{aligned} \right. \end{aligned}$$

Then, from (12), we derive the normalized Cauchy problem in $L^2_w(0,1)$:

13 $$\begin{aligned} \frac{\mathrm d}{\mathrm dt}\,{\tilde{y}}_n(t)=\tilde{{\mathcal {A}}}^\varepsilon (E(t;{{\varvec{u}}}))y_n(t)+g(t;E(t;{{\varvec{u}}})){{\tilde{\delta }}}_n\text { for }t\in (0,T],~{\tilde{y}}_n(0)={\tilde{y}}_0, \end{aligned}$$

where the operator $\tilde{{\mathcal {A}}}^\varepsilon (E)$, $E\in {{\mathscr {E}}_\mathsf {ad}}$, is defined as

$$\begin{aligned}&\mathrm {dom}\,\tilde{{\mathcal {A}}}^\varepsilon (E)=\big \{{{\tilde{\varphi }}}\in L^2_w(0,1)\,\big |\,{{\tilde{\varphi }}}\text { is absolutely continuous on }[0,1],\\&\quad {{\tilde{\varphi }}}(0)=0,v(E){{\tilde{\varphi }}}'-\kappa ^\varepsilon (h(\cdot );E){{\tilde{\varphi }}}\in L^2_w(0,1)\big \},\\&\quad \tilde{{\mathcal {A}}}^\varepsilon (E){{\tilde{\varphi }}}=-v(E) {{\tilde{\varphi }}}'+\kappa ^\varepsilon (h(\cdot );E){{\tilde{\varphi }}}\text { for }{{\tilde{\varphi }}}\in \mathrm {dom}\,\tilde{{\mathcal {A}}}^\varepsilon (E) \end{aligned}$$

and ${\tilde{y}}_0=y_0\circ h:[0,1]\rightarrow {\mathbb {R}}$ holds. Note that $y_n = \varXi ^{-1} {\tilde{y}}_n$ solves (12), where ${\tilde{y}}_n$ is the mild solution of the Cauchy problem (13).

### Approximation of the abstract Cauchy problems

In the following we derive a discretization of the Cauchy problem (13) based on shifted Legendre polynomials. This approach was originally presented in Kappel and Zhang (1993) where they apply it to a very similar type of equation. In the considered 1D case only few basis elements are needed which yields a fast approximation. However, there are situations where one would expect diffculties due to the (oscillating) characteristics of Legendre polynomials. Higher spatial dimension of the hyperbolic equation or large maturation velocities are examples for such situations. In the following, we first recall certain characteristics and further utilized features of Legendre polynomials. For more details we refer the reader to the book by Abramowitz and Stegun (1970).

**Legendre polynomials** Legendre polynomials $L_{j},\, j\in {\mathbb {N}},$ are orthogonal polynomials on $[-1,1]$ with

$$\begin{aligned} \int \limits _{-1}^{1}L_{j}(x)L_{k}(x)dx={\left\{ \begin{array}{ll} 2/(2j+1) &{} \text {for }j=k,\\ 0 &{} \text {otherwise. } \end{array}\right. } \end{aligned}$$

The polynomials can be computed with Bonnet’s recursion formula

14 $$\begin{aligned} (j+1)L_{j+1}(x)=(2j+1)xL(x)-jL_{j-1}(x),\, j=2,3,4,\dots , \end{aligned}$$

where $L_{0}(x)=1,$ and $L_{1}(x)=x.$ Thus, the first five Legendre polynomials are of the following form

$$\begin{aligned} L_{0}(x)&=1,\\ L_{1}(x)&=x,\\ L_{2}(x)&=\frac{1}{2}(3x^{2}-1),\\ L_{3}(x)&=\frac{1}{2}(5x^{3}-3x),\\ L_{4}(x)&=\frac{1}{8}(35x^{4}-30x^{2}+3). \end{aligned}$$

To compute the approximations for the partial differential equations we use the following formula representing the derivatives of Legendre polynomials in terms of Legendre polynomials:

15 $$\begin{aligned} L_{j}^{\prime }(x)={\left\{ \begin{array}{ll} \sum _{\nu =0}^{m-1}(4\nu +3)L_{2\nu +1}(x) &{} \text {for }k=2m,\\ \sum _{\nu =0}^{m}(4\nu +1)L_{2\nu }(x) &{} \text {for }k=2m+1. \end{array}\right. } \end{aligned}$$

Furthermore, the following consequences from Bonnet’s formula (eq. (14)) are needed for the approximation:

$$\begin{aligned} xL_{j}(x)= & {} \frac{j+1}{2j+1}L_{j+1}+\frac{j}{2j+1}L_{j-1},\quad j\in {\mathbb {N}},\\ x^{2}L_{j}(x)= & {} \frac{(j+1)(j+2)}{(2j+1)(2j+3)}L_{j+2}+\left( \frac{(j+1)^{2}}{(2j+1)(2j+3)}+\frac{j^{2}}{(2j-1)(2j+1)}\right) L_{j}\\&\quad +\,\frac{j(j-1)}{(2j-1)(2j+1)}L_{j-2},\quad j\in {\mathbb {N}}, \end{aligned}$$

where we set $L_{-1}(x)\equiv L_{-2}(x)\equiv 0.$

Another property of the Legendre polynomials which is used is:

16 $$\begin{aligned} \begin{aligned} L_{j}(1)&=1\\ L_{j}(-x)&=(-1)^{j}L_{j}(x),\quad j\in {\mathbb {N}}. \end{aligned} \end{aligned}$$

**Approximation** Let us define the basis functions

$$\begin{aligned} e_j(\xi ) =\frac{1}{\sqrt{w}}\,L_j(-1 + 2 \xi ), \quad 0 \le \xi \le 1, \quad j\in {\mathbb {N}}. \end{aligned}$$

The sequence $\{e_j\}_{j\in \mathbb {\mathbb {N}}}$ is an orthogonal sequence in $L_w^2(0,1)$ with

17 $$\begin{aligned} {\langle e_j,e_k\rangle }_w=0\text { for }j\ne k\quad \text {and}\quad {\Vert e_j\Vert }_w^2={\langle e_j,e_j\rangle }_w=\frac{1}{2j+1}. \end{aligned}$$

For $N\in \mathbb {\mathbb {N}}$ we introduce the *N*-dimensional subspace $X_N \subset L^2_w(0,1)$ by

$$\begin{aligned} X_N = {{\,\mathrm{span}\,}}{\big \{e_0,\ldots , e_{N-1}\big \}}. \end{aligned}$$

Let ${\mathcal {P}}_N:L_w^2(0,1)\rightarrow X_N$ be the orthogonal projection defined as

$$\begin{aligned} {\mathcal {P}}_N{{\tilde{\varphi }}} =\sum _{j=0}^{N-1}\frac{{\langle {{\tilde{\varphi }}},e_j\rangle }_w}{\Vert e_j\Vert _w^2}\,e_j\quad \text {for }{{\tilde{\varphi }}}\in L^2_w(0,1). \end{aligned}$$

The approximating delta distribution ${{\tilde{\delta }}}_N$ on $X_N$ is defined by

18 $$\begin{aligned} {\langle {{\tilde{\delta }}}_N,{{\tilde{\varphi }}}\rangle }_w={{\tilde{\varphi }}} (0)\quad \text {for }{{\tilde{\varphi }}}\in X_N. \end{aligned}$$

For any $E\in {{\mathscr {E}}_\mathsf {ad}}$ we define the approximating linear operator $A^\varepsilon _N(E):X_N\rightarrow X_N$ by

$$\begin{aligned} {\tilde{A}}^\varepsilon _N(E)\varphi =-\frac{v(E)}{w}\,\varphi '+{\mathcal {P}}_N\big (\kappa ^\varepsilon (h(\cdot );E)\varphi \big )-{{\tilde{\delta }}}_N\varphi (0)\quad \text {for }\varphi \in X_N. \end{aligned}$$

Note that the projection ${\mathcal {P}}_N$ needs only be applied on $\kappa ^\varepsilon \big (h(\cdot );E\big ) \varphi$ in case of the erythrocytes model equation due to $\varphi '\in X_N$ for $\varphi \in X_N$. Now, the discretization of (13) is given as

19 $$\begin{aligned} \begin{aligned} \frac{\mathrm d}{\mathrm dt}\,{\tilde{y}}_N(t)&={\tilde{A}}^\varepsilon _N(E(t;{{\varvec{u}}})){\tilde{y}}_N(t)+g(t;E(t;{{\varvec{u}}})) {{\tilde{\delta }}}_N\quad \text {for }t\in (0,T], \\ {\tilde{y}}_N(0)&={\mathcal {P}}_N{\tilde{y}}_0. \end{aligned} \end{aligned}$$

Let

$$\begin{aligned} y_N(t,\cdot )=\varXi ^{-1}\big ({\tilde{y}}_N(t,\cdot )\big )={\tilde{y}}_N(t,h^{-1} (\cdot )):\varOmega \rightarrow {\mathbb {R}},\quad t\in [0,T], \end{aligned}$$

where ${\tilde{y}}_N$ is a solution to (19). It follows that $\lim _{N\rightarrow \infty }y_N(t)=y(t)$ in $L^2(\varOmega )$ for $t\in [0,T]$. A proof for the case $g\equiv 0$ can be found in Kappel and Zhang (1993, Theorem 4.3). Using (17), (18) and

$$\begin{aligned} {\tilde{y}}_N(t,\xi ) = \sum _{j=0}^{N-1}{{\tilde{\mathrm {y}}}}_j(t)e_j(\xi ),\quad (t,\xi )\in [0,T]\times [0,1], \end{aligned}$$

we derive the Galerkin scheme

20 $$\begin{aligned} \begin{aligned} {\Vert e_i\Vert }_w^2\,\frac{\mathrm d}{\mathrm dt}\,{{\tilde{\mathrm {y}}}}_i(t)&=\sum _{j=0}^{N-1}{{\tilde{\mathrm {y}}}}_j(t){\langle A^\varepsilon _N(E(t;{{\varvec{u}}}))e_j,e_i\rangle }_w+g(t;E(t;{{\varvec{u}}}))e_i(0),\\ {{\tilde{\mathrm {y}}}}_i(0)&=\frac{{\langle {\tilde{y}}_0,e_i\rangle }_w}{\Vert e_i\Vert _w^2} \end{aligned} \end{aligned}$$

for $$i=0,\ldots ,N-1$$. Setting

$$\begin{aligned}&{\tilde{\varvec{y}}}(t)=\big ({{\tilde{\mathrm {y}}}}_{i-1}(t)\big )_{1\le i\le N}\qquad \text {for }t\in [0,T],\\&{\varvec{A}}^\varepsilon (E)=\bigg (\frac{\langle A^\varepsilon _N(E)e_{j-1},e_{i-1}\rangle _w}{\Vert e_{i-1}\Vert _w^2}\bigg )_{1\le i,j\le N}\qquad \text {for }E\in {{\mathscr {E}}_\mathsf {ad}},\\&{\varvec{d}}=\bigg (\frac{e_{i-1}(0)}{\Vert e_{i-1}\Vert _w^2}\bigg )_{1\le i\le N},\quad {\tilde{\varvec{y}}}_0=\bigg (\frac{{\langle {\tilde{y}}_0,e_{i-1} \rangle }_w}{\Vert e_{i-1}\Vert _w^2}\bigg )_{1\le i\le N} \end{aligned}$$

we can express (20) as the following system of *N* ordinary differential equations

21 $$\begin{aligned} \frac{\mathrm d}{\mathrm dt}\,{\tilde{\varvec{y}}}(t)={\varvec{A}}^\varepsilon (E(t;{{\varvec{u}}})) {\tilde{\varvec{y}}}(t)+g(t;E(t;{{\varvec{u}}})){\varvec{d}}\text { for }t\in (0,T],\quad {\tilde{\varvec{y}}}(0)={\tilde{\varvec{y}}}_0. \end{aligned}$$

#### Theorem 1

Let $E(\cdot ;{{\varvec{u}}})$ given by (4) for an arbitrarily chosen ${{\varvec{u}}}\in {{\mathscr {U}}_\mathsf {ad}}$. Moreover, $g(\cdot ;E):[0,T]\rightarrow {\mathbb {R}}$ is assumed to be piecewise continuous for every $E\in {{\mathscr {E}}_\mathsf {ad}}$. Then, the function

22 $$\begin{aligned} {\tilde{\varvec{y}}}(t)=e^{\int _0^t{\varvec{A}}^\varepsilon (E(s;{{\varvec{u}}}))\,\mathrm {d}s}{\tilde{\varvec{y}}}_0+ \bigg (\int _0^te^{\int _\tau ^t{\varvec{A}}^\varepsilon (E(s;{{\varvec{u}}}))\,\mathrm {d}s}g(\tau ;E(\tau ;{{\varvec{u}}}))\,\mathrm {d}\tau \bigg ){\varvec{d}} \end{aligned}$$

is the only one that satisfies (21) at every time instance, where $g(\cdot ;E)$ is continuous for every $E\in {{\mathscr {E}}_\mathsf {ad}}$. Furthermore, ${\tilde{\varvec{y}}}\in H^1(0,T;{\mathbb {R}}^N)$ holds.

#### Proof

1. We first show that (22) satisfies (21). Using $E_\mathrm {min}>0$ it follows from Lemmas 1-3 that the mappings $t\mapsto v_i(E(t;{{\varvec{u}}}))$, and $t\mapsto \kappa _i^\varepsilon (x;E(t;{{\varvec{u}}}))$, $1 \le i \le 5$, are continuously differentiable on [0, *T*] for every $x\in \varOmega$. Hence, the mapping ${\varvec{A}}^\varepsilon (E(\cdot ;{{\varvec{u}}})):[0,T]\rightarrow {\mathbb {R}}^{N\times N}$ is continuously differentiable for every ${{\varvec{u}}}\in {{\mathscr {U}}_\mathsf {ad}}$. We obtain for any $t\in (0,T]$, where $g(\cdot ;E)$ is continuous
  $$\begin{aligned} \frac{\mathrm {d}}{\mathrm {d}t}{\tilde{\varvec{y}}}(t)&={\varvec{A}}^\varepsilon (E(t;{{\varvec{u}}})) e^{\int _0^t{\varvec{A}}^\varepsilon (E(s;{{\varvec{u}}}))\,\mathrm {d}s}{\tilde{\varvec{y}}}_\circ +g(t;E(t;{{\varvec{u}}})){\varvec{d}} \\&\quad +{\varvec{A}}^\varepsilon (E(t;{{\varvec{u}}}))\bigg (\int _0^te^{\int _\tau ^t{\varvec{A}}^\varepsilon (E(s;{{\varvec{u}}}))\,\mathrm {d}s}g(\tau ;E(\tau ;{{\varvec{u}}}))\,\mathrm {d}\tau \bigg ){\varvec{d}}\\&={\varvec{A}}^\varepsilon (E(t;{{\varvec{u}}})){\tilde{\varvec{y}}}(t)+g(t;E(t;{{\varvec{u}}})) {\varvec{d}}. \end{aligned}$$
  Further, if we choose $t=0$ in (22) we get ${\tilde{\varvec{y}}}(0)={\tilde{\varvec{y}}}_0$. Hence, ${\tilde{\varvec{y}}}$ satisfies (21) at every time instance, where $g(\cdot ;E)$ is continuous.
2. Uniqueness: Assume there exist two solutions ${\tilde{\varvec{y}}}^1$, ${\tilde{\varvec{y}}}^2$ to (21). We set ${\tilde{\varvec{z}}}={\tilde{\varvec{y}}}^1-{\tilde{\varvec{y}}}^2$. Then, it follows that
  $$\begin{aligned} \frac{\mathrm {d}}{\mathrm {d}t}\,{\tilde{\varvec{z}}}(t)={\varvec{A}}^\varepsilon (E(t;{{\varvec{u}}})){\tilde{\varvec{z}}}(t) \end{aligned}$$
  for all $t\in (0,T]$, where $g(\cdot ;E)$ is continuous. Since ${\varvec{A}}^\varepsilon (E(\cdot ;{{\varvec{u}}}))$ and ${\tilde{\varvec{z}}}$ are continuous, we can extend the derivative of ${\tilde{\varvec{z}}}$ by ${\varvec{A}}^\varepsilon (E(t;{{\varvec{u}}})){\tilde{\varvec{z}}}(t)$ for all $t\in (0,T]$. Furthermore, we have ${\tilde{\varvec{y}}}^1(0)={\tilde{\varvec{y}}}^2(0)$. From Gronwall’s inequality we infer that ${\tilde{\varvec{z}}}(0)=0$ in [0, *T*], which implies ${\tilde{\varvec{y}}}^1={\tilde{\varvec{y}}}^2$ in [0, *T*].

$$\square $$

#### Remark 1

1. Due to Theorem 1 the solution
  $$\begin{aligned} {\tilde{y}}_N(t,\xi ) = \sum _{j=0}^{N-1}{{\tilde{\mathrm {y}}}}_j(t)e_j(\xi ),\quad (t,\xi )\in [0,T]\times [0,1], \end{aligned}$$
  to (19) belongs to $H^1(0,T;L^2_w(0,1))$ provided $g(\cdot ;E):[0,T]\rightarrow {\mathbb {R}}$ is piecewise continuous for every $E\in {{\mathscr {E}}_\mathsf {ad}}$.
2. To solve (21) a time integration method has to be applied. In our numerical experiments we apply the implicit Euler method.$\Diamond$

### The control-to-state operator

Let ${{\varvec{u}}}\in {{\mathscr {U}}_\mathsf {ad}}$ be a given control variable and $E(\cdot ;{{\varvec{u}}})$ be given by (4). Then, the five state variables $(y_i)_{1\le i\le 5}$ solving (${\mathbf {S}}^\varepsilon$) are approximated by the functions

23 $$\begin{aligned} y_{i,N}(t,\cdot )={\tilde{y}}_{i,N}(t,h^{-1}(\cdot ))=\sum _{j=0}^N {\tilde{\mathrm {y}}}_{ij}(t)e_j(h^{-1}(\cdot ))\in L^2(\varOmega ), \end{aligned}$$

where the coefficient vector ${\tilde{\varvec{y}}}_i=({\tilde{\mathrm {y}}}_{i,j-1})_{1\le j\le N}$ satisfies 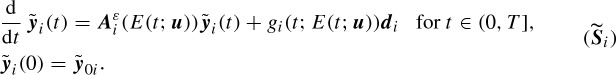 This is Eq. (21) with added index *i*. Due to Theorem 1 we define the non-linear solution operatorwhere  is the generalized Cartesian product. Hence, ${\tilde{\varvec{y}}}=({\tilde{\varvec{y}}}_i)_{1 \le i\le 5}={\mathcal {S}}_N({{\varvec{u}}})$ satisfies ($\varvec{{\widetilde{S}}_1}$)–($\varvec{{\widetilde{S}}_5}$). Then, the discretized total RBC population is given by (cf. (7))

24 $$\begin{aligned} P_N[{\tilde{\varvec{y}}}](t)=\sum _{j=0}^N{\tilde{\mathrm {y}}}_{5,j}(t)\int _{\varOmega _5} e_j(h^{-1}(x))\,\mathrm {d}x= {\tilde{\mathrm {y}}}_{5,0}(t)\, \omega _5^{1/2}\quad \text {for }t\in [0,T], \end{aligned}$$

where again ${\tilde{\varvec{y}}}=({\tilde{\varvec{y}}}_i)_{1 \le i\le 5}$ satisfies ($\varvec{{\widetilde{S}}_1}$)–($\varvec{{\widetilde{S}}_5}$). Note that the equations (${\widetilde{\varvec{S}}_i}$), $i=1,2,\ldots ,5$ can be solved consecutively. At any time $t \in [0,T]$ the discrete state vector ${\tilde{\varvec{y}}}(t)$ is of length 5*N*.

## The optimal EPO dosing

In this section we formulate the optimal EPO dosing as an optimal control problem on the long time horizon [0, *T*]. For the number $$n_u\in {\mathbb {N}}$$ let the days $$\{t_u^j\}_{j=1}^{n_u+1}$$ with a constant EPO rate in $$[t_u^j,t_u^{j+1})$$, $$j=1,\ldots ,n_u$$, be given as

$$\begin{aligned} 0 = t_u^1<\cdots <t_u^{n_u+1} = T. \end{aligned}$$

### The optimal control problem

The goal is to stabilize the Hgb around a desired value of 10.5 g/dl in order to bring and keep the Hgb into the target window of 10–12 g/dl. Values in this range are considered as safe. We choose this lower value within the range because in case of an overshoot the Hgb can not be pulled down actively but one has to wait till it decreases of itself. The optimal control problem is formulated for the number of RBCs and not for the Hgb. This means that, for each patient, prior to optimization a desired total amount $P^d$ of RBCs is calculated using formula (8).

In the cost functional, we measure and penalize the deviation of the total RBC population from the desired population $P^d$ over the whole time horizon [0, *T*]

25 $$\begin{aligned} {\hat{J}}_N({{\varvec{u}}})=\frac{1}{2}\sum _{j=1}^{n_u} \gamma _j \,|u_j|^2+\frac{\sigma _\varOmega }{2}\int _0^T\big |P_N[{\tilde{\varvec{y}}}](t)-P^d\big |^2\,\,\mathrm {d}t, \end{aligned}$$

where the discretized total RBC population $P_N[{\tilde{\varvec{y}}}]$ has been introduced in (24) and ${\tilde{\varvec{y}}}={\mathcal {S}}_N({{\varvec{u}}})$ satisfies ($\varvec{{\widetilde{S}}_1}$)–($\varvec{{\widetilde{S}}_5}$). Further, $\gamma _1,\ldots ,\gamma _{n_u}>0$ are regularization parameters and $\sigma _\varOmega$ is a non-negative weight. Now, the optimal control problem is formulated as follows: 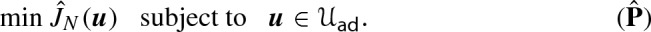


#### Remark 2

1. Note that (${{\hat{\mathbf{P}}}}$) is a non-linear optimization problem. Hence, (${{\hat{\mathbf{P}}}}$) is non-convex, so that several local minima might exist.$\Diamond$
2. It can be shown that (${{\hat{\mathbf{P}}}}$) possesses at least one optimal control in ${{\mathscr {U}}_\mathsf {ad}}$. For the sake of brevity, we do not present the proof here.

### The NMPC method

Problem (${{\hat{\mathbf{P}}}}$) can not be treated as an open-loop problem since unforeseen events and disturbances can occur. In reality, predicted and measured Hgb values will differ which has to be taken into account by means of a closed-loop controller. Moreover, patient parameters will not be constant over time which even makes readaptations of the model necessary in actual application.

Let all time intervals of constant administration rates be of same length $\varDelta _t>0$ and

$$\begin{aligned} T=L\varDelta _t\quad \text {for }L\in {\mathbb {N}}. \end{aligned}$$

Suppose we are at time ${t_\circ }\in [0,T-M\varDelta _t]$ and consider the time horizon $[{t_\circ },{t_{\mathsf {f}}}]$ with ${t_{\mathsf {f}}}={t_\circ }+M\varDelta _t$, $M\in {\mathbb {N}}$ and $M\varDelta _t\ll T$.

Next we introduce a notation for the days

$$\begin{aligned} \{t_u^{j_l}\}_{l=1}^{n({t_\circ })+1}\subset \{t_u^1,\ldots ,t_u^{n_u+1}\}\subset [0,T]\quad \text {with}\quad {t_\circ }\le t_u^{j_1}<\cdots <t_u^{j_{n_u({t_\circ })+1}}\le {t_{\mathsf {f}}}, \end{aligned}$$

which belong to the current horizon $[{t_\circ },{t_{\mathsf {f}}}]$; cf. (2). At ${t_\circ }$ we are given the initial conditions ${\tilde{\varvec{y}}}_\circ =({\tilde{\varvec{y}}}_{\circ i})_{1\le i\le 5}$ and $E^\mathrm {ex}({t_\circ }) = E^\mathrm {ex}_\circ$. Then, we define the cost functional on the time horizon $[{t_\circ },{t_{\mathsf {f}}}]\subset [0,T]$ with $0\le {t_\circ }<{t_{\mathsf {f}}}$ and ${t_{\mathsf {f}}}-{t_\circ }\ll T$:

26 $$\begin{aligned} \begin{aligned} {\hat{J}}_N({{\varvec{u}}};{t_\circ },{\tilde{\varvec{y}}}_\circ ,E^\mathrm {ex}_\circ )&=\frac{1}{2}\sum _{l=1}^{n_u({t_\circ })} \gamma _{j_l}\,|u_{j_l}|^2+\frac{\sigma _\varOmega }{2}\int _{t_\circ }^{t_{\mathsf {f}}}\big |P_N[{\tilde{\varvec{y}}}](t)-P^d\big |^2\,\,\mathrm {d}t\\&\quad +\frac{\sigma _{\mathsf {f}}}{2}\,\big |P_N[{\tilde{\varvec{y}}}]({t_{\mathsf {f}}})-P^d\big |^2, \end{aligned} \end{aligned}$$

where the coefficient vector ${\tilde{\varvec{y}}}=({\tilde{\varvec{y}}}_i)_{1 \le i\le 5}$ satisfies ($\varvec{{\widetilde{S}}_1}$)–($\varvec{{\widetilde{S}}_5}$) on the time horizon $[{t_\circ },{t_{\mathsf {f}}}]$ with initial conditions ${\tilde{\varvec{y}}}_{\circ i}$, $1\le i\le 5$. In (26) we have added a summand penalizing the deviation of the population at the final time ${t_{\mathsf {f}}}$ for stability reasons. For ${\mathscr {U}}(t_\circ )={\mathbb {R}}^{n_u({t_\circ })}$ we define the admissible set

$$\begin{aligned} {{\mathscr {U}}_\mathsf {ad}}({t_\circ })=\big \{{{\varvec{u}}}\in {\mathscr {U}}(t_\circ )\,\big |\,0\le {{\varvec{u}}}\le u_\mathsf {max}\big \}. \end{aligned}$$

Considering a constant EPO rate per day in our numerical experiments we set $\gamma _j \equiv \frac{c_\gamma }{M\varDelta _t}$ for some constant $c_\gamma > 0$. Note that $M\varDelta _t$ is the length of the prediction horizon. Now, the controller predicts the future evolution of the system under control over this prediction horizon, the cost functional ${\hat{J}}_N({{\varvec{u}}};{t_\circ },{\tilde{\varvec{y}}}_{\circ },E^\mathrm {ex}_\circ )$ gets minimized and we obtain an optimal control vector for the given time period. Then, (only) the first component of the optimal control vector is applied and yields new initial conditions for the next initial time point ${t_\circ }+\varDelta _t$, to where the finite horizon gets pushed. In Algorithm 1 we summarize the NMPC method. The iterative computation of the control ${{\varvec{u}}}$ can be seen in line 5 where in each iteration the first component of the optimal open loop solution to problem (${\hat{\mathbf{P}}}({t_\circ })$) from line 4 is appended to the already given control vector. Then, this control is applied on the corresponding time interval $[{t_\circ },{t_\circ }+ \varDelta _t]$ and the initial condition for the next iteration is set to ${\tilde{\varvec{y}}}({t_\circ }+ \varDelta _t)$; see line 6. If the state vectors can be measured or estimated based on measurements, the new initial condition ${\tilde{\varvec{y}}}_\circ$ in line 6 can be set therewith.
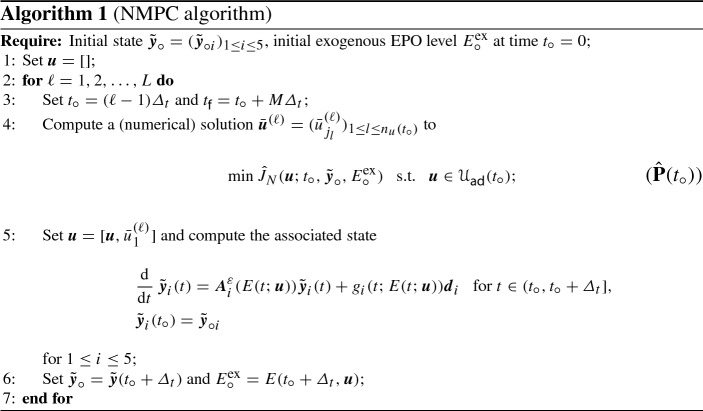


### Numerical solution of the open-loop problem (${{\hat{\mathbf{P}}}}({t_\circ })$)

It remains to discuss how the open-loop problem in line 4 of Algorithm 1 is numerically solved. In this work we apply a projected BFGS method with Armijo linesearch as described in Kelley (1999, Sect. 5.5.3). This requires to compute the gradient $\nabla {\hat{J}}_N=(\partial _{u_{j_l}}{\hat{J}}_N)_{1\le l\le n_u({t_\circ })}$ at ${{\varvec{u}}}=(u_{j_l})_{1\le l\le n_u({t_\circ })}\in {{\mathscr {U}}_\mathsf {ad}}({t_\circ })$. This is done using a Lagrangian-based approach as shown in Hinze et al. (2009)[Sect. 1.6.4]. The gradient is given by

$$\begin{aligned}&\partial _{u_{j_l}}{\hat{J}}_N({{\varvec{u}}};{t_\circ },{\tilde{\varvec{y}}}_\circ ,E^\mathrm {ex}_\circ )=\gamma _{j_l}u_{j_l}\\&\quad +\sum _{i=1}^5\int _{t_\circ }^{t_{\mathsf {f}}}\bigg (\frac{\partial E}{\partial u_{j_l}}(t;{{\varvec{u}}})\Big (\frac{\partial {\varvec{A}}^\varepsilon _i}{\partial u_{j_l}}(E(t;{{\varvec{u}}})){\tilde{\varvec{y}}}_i(t)-\frac{\partial g_i}{\partial u_{j_l}}(E(t;{{\varvec{u}}})){\varvec{d}}_i\Big )\bigg )^\top {\varvec{D}}_\omega {\tilde{\varvec{p}}}_i(t)\,\mathrm {d}t, \end{aligned}$$

with the adjoint variable ${\tilde{\varvec{p}}}=({\tilde{\varvec{p}}}_i)_{1\le i\le 5}$, where ${\tilde{\varvec{p}}}_i$ satisfies 
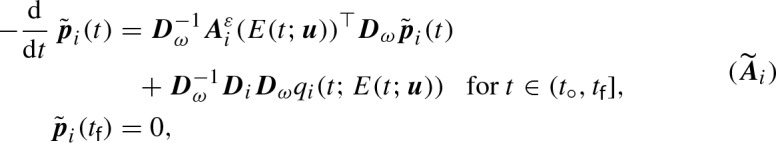


for $i=1,\ldots ,5$, with ${\varvec{D}}_\omega = \mathrm {diag}(\frac{1}{2j+1}\mid j = 0,\ldots ,N)$, ${\varvec{D}}_i = \mathrm {diag}({\varvec{d}}_i)$, $i = 1,\ldots ,5$ and

$$\begin{aligned} q_i(t;E)=\left\{ \begin{aligned}&\omega _{i+1}^{-1/2} {\tilde{\varvec{p}}}_{i+1}(t)&\text {if }i=1,2,\\&\omega _{4}^{-1/2}{\tilde{\varvec{p}}}_{4}(t)\big /\nu (E)&\text {if }i=3,\\&\omega _{5}^{-1/2}\nu (E){\tilde{\varvec{p}}}_{5}(t)&\text {if }i=4. \end{aligned} \right. \end{aligned}$$

Note that the adjoint state ${\tilde{\varvec{p}}}$ is the Lagrange multiplier associated with the state equation.

We have presented the gradient and the adjoint equations ($\varvec{{\widetilde{A}}_1}$)–($\varvec{{\widetilde{A}}_5}$) in a very compact format. So let us explain what it actually means to calculate the gradient $\nabla {\hat{J}}_N$ at ${{\varvec{u}}}=(u_{j_l})_{1\le l\le n_u({t_\circ })}\in {{\mathscr {U}}_\mathsf {ad}}({t_\circ })$:

1. Solve the state equations ($\varvec{{\widetilde{S}}_1}$)–($\varvec{{\widetilde{S}}_5}$) on the time horizon $[{t_\circ },{t_{\mathsf {f}}}]$ forward in time.
2. Solve the adjoint state equations ($\varvec{{\widetilde{A}}_5}$)–($\varvec{{\widetilde{A}}_1}$) on the time horizon $[{t_\circ },{t_{\mathsf {f}}}]$ backward in time.
3. Compute the gradient $\nabla {\hat{J}}_N$.

## Numerical results

For our numerical experiments we have chosen the data sets from five patients which capture the main occurring characteristics. The constant endogenous erythropoietin concentration $E^\mathrm {end}$ for example is once far above the threshold for neocytolysis ($\tau _E = 80$), twice just slightly below and twice clearly smaller. The aim was to find a general optimization setting that works for diverse data sets. The parameters can be looked up in Table 8.

For computation of the population densities we scale the hyperbolic equations by $10^8$ which is legitimate since the equations are linear with respect to the state variables $y_i$, $i = 1, \ldots , 5$. For discretization we use $N=15$ Legendre polynomials and the time step size $\varDelta t = 0.01$.

We first have a look at the predicted Hgb curves when no EPO is administered. Then, we show how the NMPC algorithm is able to correct anemia under the assumption of knowing the true model and patient parameters and being able to continuously and perfectly measure Hgb.

### Uncontrolled Hgb concentration

**Fig. 3:**
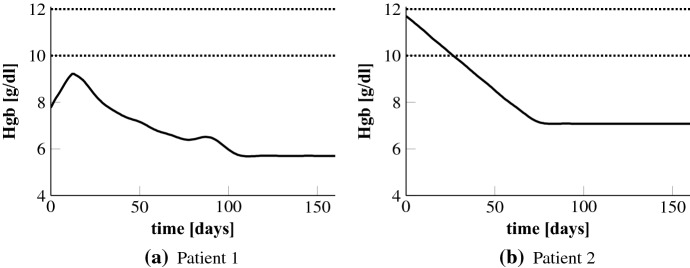
Uncontrolled Hgb levels. The dotted lines mark the target range of 10–12 g/dl

We begin by taking a look at the predicted Hgb concentrations without EPO administration. As can be seen exemplarily in Fig. 3 the Hgb levels are running in a steady state far below the target range. This state of anemia would be critical for patients since it increases cardiovascular disease and death risk (Strippoli et al. 2004).

### NMPC

#### Settings

Throughout we consider a total time period of 24 weeks, i.e. $T = 168$ days. Excluding our last experiment, the days $\{t_u^j\}_{j=1}^{n_u+1}$ are given by $\{0,1,\ldots ,168\}$ and the length of the NMPC horizon is four weeks. This means, we set $\varDelta _t = 1$, $M = 28$ and $L = 168$. Note that the length of the horizon determines the computational effort of the NMPC method: the longer the horizon, the more time it takes to calculate the numerical solution to the open-loop problem in each step. Hence, one is searching for the minimal horizon that provides stability of the NMPC closed-loop. In our simulations we have observed that four weeks serves the purpose for the named setting. The fact that the cells remain about two weeks in the bone marrow (first four classes) before released into the bloodstream explains the need for this length. In pratice, the maximum cumulative weekly EPO dose is 60,000 U with up to three administrations a week. Given our higher administration frequency we set the maximum weekly EPO dose to 7000 U which leads us to set $u_\mathrm {max} = 1000$ U/day.

Given the time horizon $M\varDelta _t$ we set

27 $$\begin{aligned} \sigma _\varOmega = \frac{10^{4}}{M\varDelta _t r^2},\quad \sigma _{\mathsf {f}} = \frac{10^{3}}{r^2}, \quad r = \frac{2\,\mathrm {MCH}}{c_\mathsf {tbv} 10^{10}}, \quad \gamma _j \equiv \frac{c_\gamma }{M\varDelta _t}, \end{aligned}$$

where only the parameter $c_\gamma$ is chosen in an individualized manner.

**Fig. 4:**
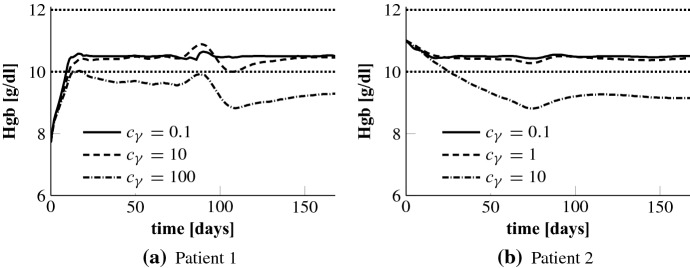
Optimal Hgb curves for different values $c_\gamma$ for patients 1 and 2. The dotted lines mark the target range

**Fig. 5:**
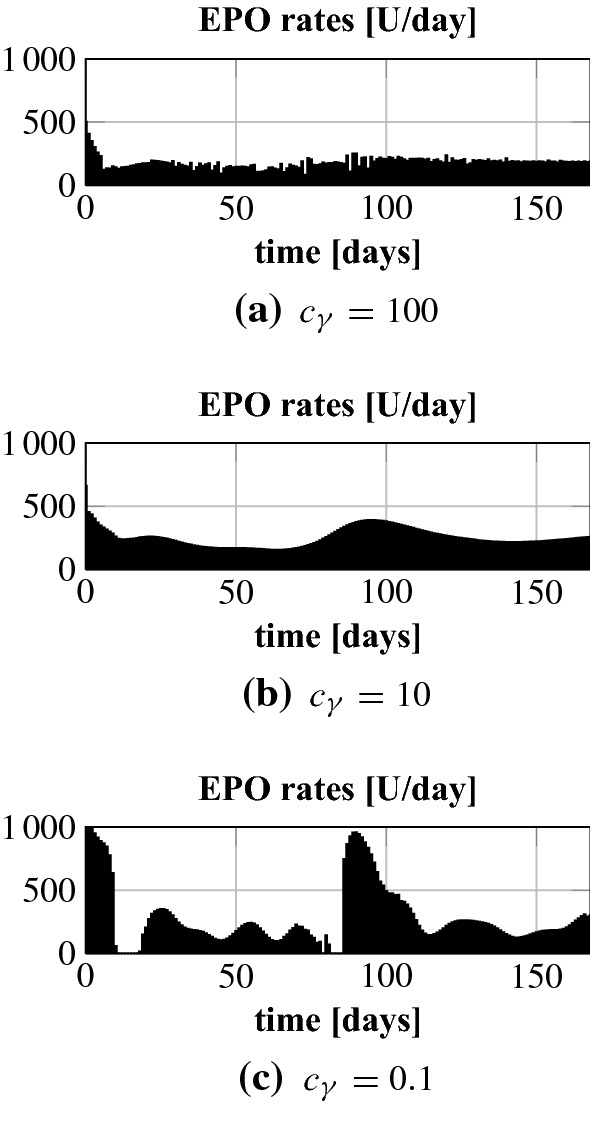
Patient 1: optimal EPO rates for different values $c_\gamma$

**Fig. 6:**
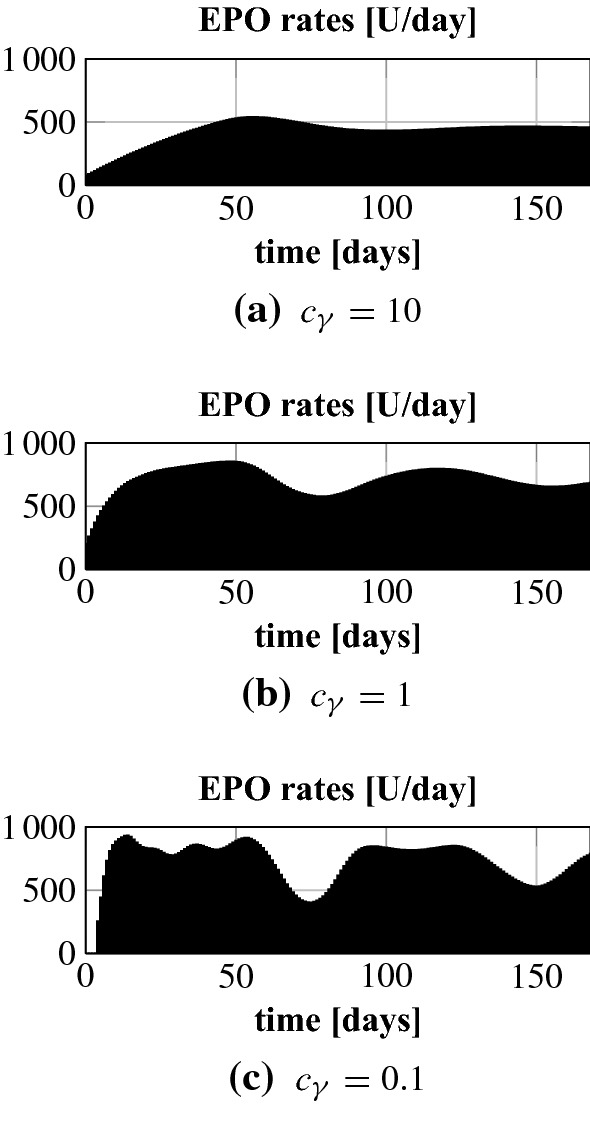
Patient 2: optimal EPO rates for different values $c_\gamma$

We start by testing the NMPC algorithm for different constants $c_\gamma$. This parameter penalizes control costs in the objective functional. A larger $c_\gamma$ results in a stronger penalization of control costs, i.e. the controller must try to bring and keep the Hgb curve in the target range with less EPO. In Fig. 4 we present the results for patients 1 and 2. For both patients the value $c_\gamma = 0.1$ is the right choice for control costs penalization in the sense that it allows to accurately stabilize the Hgb around 10.5 g/dl. The corresponding EPO rates can be seen in Figs. 5 and 6. Interestingly, the smallest penalization in patient 1 results in a very specific and time sensitive administration pattern. Patient 2 requires a lot higher doses than patient 1. It is actually about 2.5 times as much; see Table 1. When looking at the uncontrolled Hgb levels in Fig. 3 patient 2 shows a higher asymptotic Hgb level than patient 1. So the higher doses are actually counterintuitive which underlines the need for a closer look into the time dynamics. For $c_\gamma = 10$ the Hgb concentration for patient 1 is still within the target range while this penalization results in a drop below the target range for patient 2. Still, for $c_\gamma = 1$ the total population for patient 2 remains as well within the target range. Using $c_\gamma = 100$ for patient 1 leads to a Hgb level below the target range. This indicates that the penalization of the control costs needs to be chosen carefully on a subgroup or even per-patient level.

**Table 1:** Total EPO doses for different values $c_\gamma$ for patients 1 and 2

	Patient 1			Patient 2		
$c_\gamma$	0.1	10	100	0.1	1	10
Total dose (U)	48,323	42,683	30,616	121,354	119,114	71,706

Our primary goal is *Hgb in target*. Thus, we choose $c_\gamma$ such that the penalization of control costs does not block the NMPC algorithm from controlling the Hgb levels into the target range. The above test we have also done for the other patients. For patient 5 the value $c_\gamma = 0.1$ is fine as well while patients 3 and 4 require a weaker penalization of $c_\gamma = 0.01$. Concluding, with $c_\gamma = 0.01$ the Hgb levels of all patients can be brought into target only that for patients 1,2 and 5 this can as well be achieved with $c_\gamma = 0.1$ and consequently a lower total EPO dose.

#### Bleeding

**Fig. 7:**
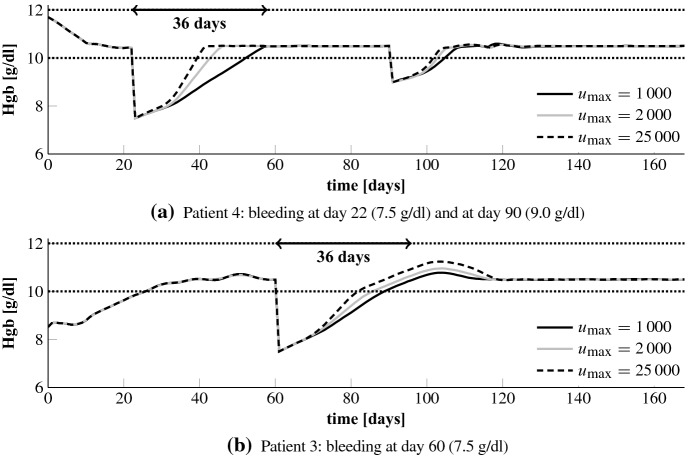
Bleeding patients 3 and 4. Up to the first bleeding $u_\mathrm {max}$ equals 1000. The dotted lines mark the target range

In the following we analyze how the NMPC algorithm handles a sudden and unforeseen (gastro-intestinal) bleeding. This is a frequent complication in HD patients. Note that the NMPC algorithm learns about the bleeding first after it has happened by an assumed measurement. In line 6 of Algorithm 1 the initial condition ${\tilde{\varvec{y}}}_\circ$ is overwritten based on that measurement. The results for patients 3 and 4 for a bleeding which corresponds to a drop in Hgb down to 7.5 g/dl are shown in Fig. 7. Patient 4 has a fast recovery time so that we add a later smaller bleeding to 9.0 g/dl. According to Pottgiesser et al. (2008) the mean recovery time period after a blood donation of 550 ml is 36 days (range 20–59). This average recovery time is added as a vertical arrow to Fig. 7. We run the optimization such that we keep the maximum EPO rate at 1000 U/day or after the first bleeding we increase $u_\mathrm {max}$ to analyze by how much the recovery time could be decreased. In addition, we set $c_\gamma = 10^{-10}$ and $u_\mathrm {max} = 25,000$ U/day after the first bleeding to see if this results in an overshoot.

**Fig. 8:**
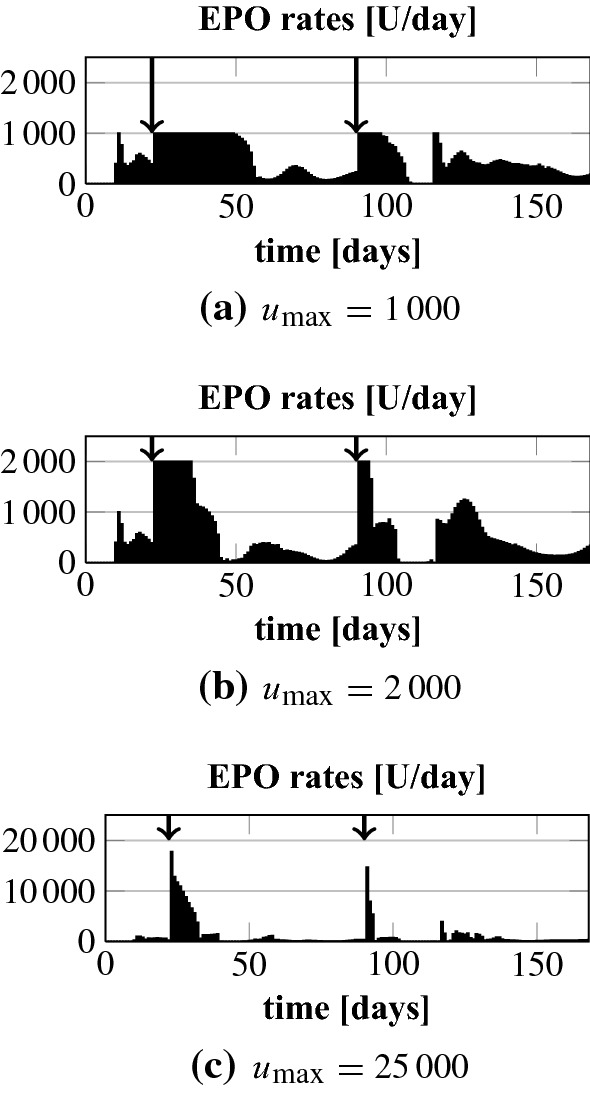
Bleeding patient 4: EPO rates for different values $u_\mathrm {max}$ after the first bleeding. Up to the first bleeding $u_\mathrm {max}$ equals 1000. The first bleeding is at day 22 (7.5 g/d) and the second at day 80 (9.0 g/d)

**Fig. 9:**
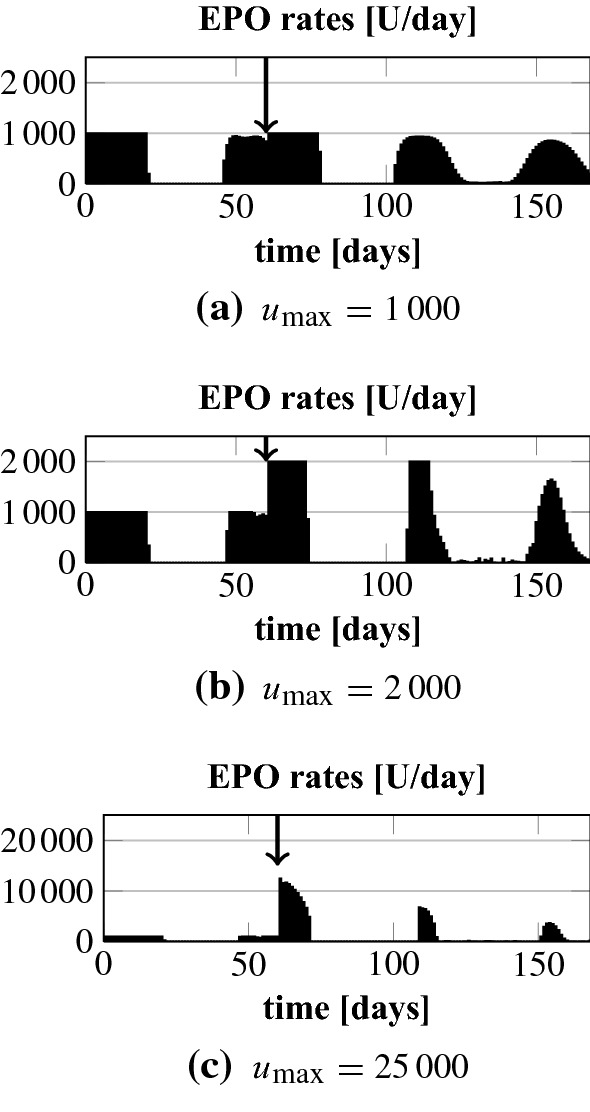
Bleeding patient 3: EPO rates for different values $u_\mathrm {max}$ after the bleeding. Up to the bleeding $u_\mathrm {max}$ equals 1000. The bleeding is at day 60 (7.5 g/d)

We first analyze the results for a constant maximum EPO rate of 1000 U/day and when that rate is increased to 2000 U/day after the first bleeding; upper plots in Figs. 8 and 9. We observe that the NMPC algorithm is reacting to the bleeding by directly administering the maximum available EPO dose over a certain period of time. For $u_\mathrm {max} = 2000$ U/day this higher amount is administered but for a shorter period of time. Hence, the total drug amount, see Table 2, is only 13% and 16% higher. The Hgb curves are shown in Fig. 7. The recovery time after the first bleeding for patient 4 can be significantly reduced from about 36 to about 25 days by allowing higher administration rates. For patient 3 the differences are minor. The reason is that a reduction of the recovery time for patient 3 comes along with an increase of the overshoot because the constant endogenous EPO concentration $E^\mathrm {end}$ for patient 3 is around 280. For patient 4 it is only 43. Hence, by reducing the administration rate for patient 4 the controller can abruptly let drop down the EPO concentration in plasma which allows a fast recovery time without any overshoot. Even for the high maximum EPO rate of 25,000 U/day together with a minor control penalization ($c_\gamma =10^{-10}$) we do not observe an overshoot in patient 4. Note that the maximum rate is never reached; see Fig. 8c. But for patient 3 the overshoot gets more pronounced while the target range is reached some days earlier; Fig. 7b. Again, the maximum rate is not hit; Fig. 9c.

**Table 2:** Bleeding patients 3 and 4: total EPO doses for different values $u_\mathrm {max}$ after the first bleeding

	Patient 4			Patient 3		
$u_\mathrm {max}$	1000	2000	25,000	1000	2000	25,000
Total dose (U)	79,124	91,853	187,660	83,980	94,948	199,077

Up to the first bleeding $u_\mathrm {max}$ equals 1000. For patient 3 the first bleeding is at day 22 (7.5 g/d) and the second at day 80 (9.0 g/d). In case of patient 3 there is only one bleeding at day 60 (7.5 g/dl)

#### Missed administrations/dosing errors

**Fig. 10:**
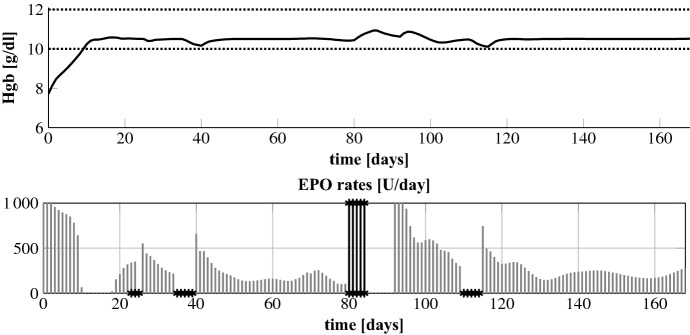
Patient 1: optimal EPO rates and Hgb curve for missed treatments on days 23–25, 35–39, 110–114 and on days 80–84 the maximum EPO rate is administered by mistake

In this section we consider a malfunction of the EPO pump. We simulate a complete failure to administer EPO for an entire day or that and incorrect rate is applied (not known in advance). In Fig. 10 we present the results for patient 1 with missed administrations on days 23–25, 35–39 and 110–114. In addition, on days 80–84 the maximum amount $u_\mathrm {max} = 1000$ U/day gets administered by mistake. Missing an administration leads to a certain direct drop in the Hgb level. After every period of missed administrations the algorithm compensates for these by applying a higher EPO rate which is then gradually reduced in order to smoothly reincrease the Hgb concentration. After the period when the maximum rate is wrongly applied the algorithm administers nothing so that the Hgb level decreases and that an overshoot is avoided. But interestingly, it then restarts with the maximum amount before dropping on the desired level and a minor reincrease is even accepted. In doing so it achieves to balance the delayed effect of having administered no drug before and a later drastic drop-down is avoided. The total administered EPO dose is 50,853 U.

**Fig. 11:**
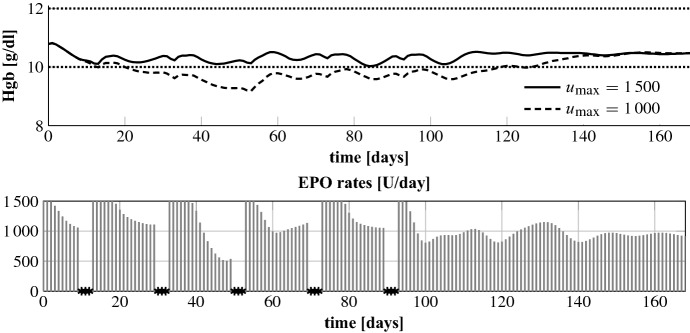
Patient 5: optimal EPO rates and Hgb curve for missed treatments on days 10–12, 30–32, 50–52, 70–72, 90–92 and $u_\mathrm {max} = 1500$. In addition, the optimal Hgb curve for $u_\mathrm {max} = 1000$ is shown in the upper plot

In Fig. 11 we present the results for periodically missed EPO administrations. More precisely, starting at day ten, every two weeks three days drop out. This scheme is chosen for analyzing if a periodic missing of administrations results in periodic administration rates. But as can be seen, the EPO rates after each period of missed treatments look different and the NMPC alogithm achieves to keep the Hgb level within the desired range if $u_\mathrm {max}$ is set to 1500 U/day. Patient 5 demands comparatively high EPO rates so that the Hgb concentration falls below the target range if $u_\mathrm {max}$ equals 1000 U/day. Note that the Hgb curve can be kept within the target range for $u_\mathrm {max} = 1000$ if there are no missed treatments. The total administered EPO doses belonging to Fig. 11 are 167,848 ($u_\mathrm {max} = 1500$) and 150,921 U/day ($u_\mathrm {max} = 1000$).

#### Constant EPO rates

Finally, we simulate different frequencies of rate change for the EPO pump. Note that less frequent adaptations could be a consequence of less frequent Hgb measurements. We investigate the effect of enlarging the period of a constant EPO rate from 1 day ($\varDelta _t = 1$) to 1 week ($\varDelta _t = 7$), 2 weeks ($\varDelta _t = 14$), 3 weeks ($\varDelta _t = 21$) and 4 weeks ($\varDelta _t = 28$). This requires to adjust the length of the NMPC horizon to 4 weeks (constant period 1 week), 6 weeks (constant period 2 or 3 weeks) and 8 weeks (constant period 4 weeks). In order to penalize control costs likewise for the different constant periods the parameter $c_\gamma$ in Eq. (27) gets multiplied by the number of days in the respective constant period.

**Fig. 12:**
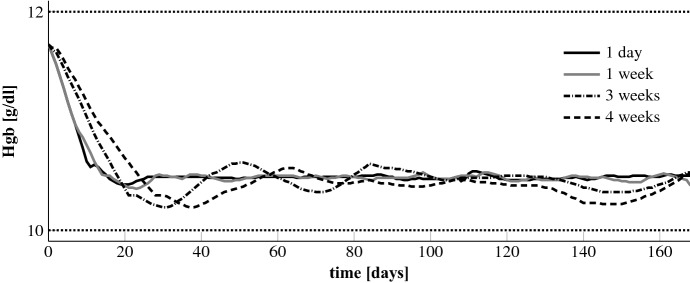
Patient 4: optimal Hgb curves for different constant periods

**Fig. 13:**
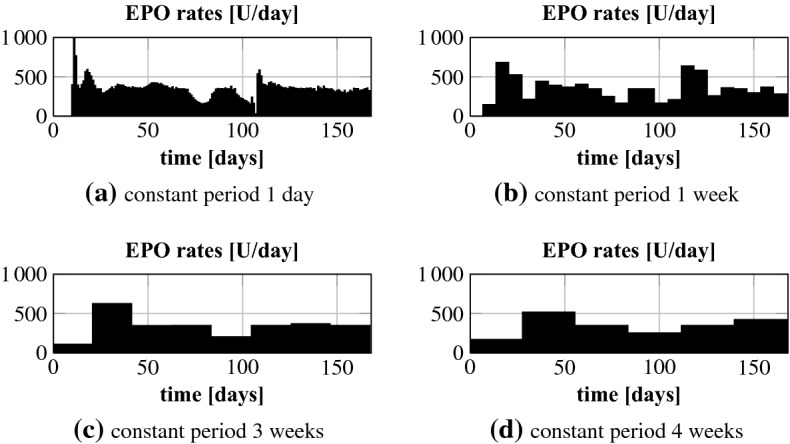
Patient 4: optimal EPO rates for different constant periods

The results for patient 4 are exemplarily shown in Figs. 12 and 13. The Hgb curves for the different constant periods are all stabilized within the target range. The same holds true for the other patients. From a mathematical point of view a longer constant period means a restriction of degrees of freedom. Therefore, the Hgb level is best controllable for the shortest constant period. This is why the Hgb level is closest to the line of 10.5 g/dl when the controller can adjust the doses on a daily basis. And the longer the constant period is, the larger are the oscillations around this line. The total EPO doses, see Table 3, differ only little. The results for patient 4 look similar to those from patient 1 which is very interesting. Figure 5 can lead one to assume that the patient requires a very specific and time sensitive administration pattern to stabilize the Hgb level. But even for a constant period of a whole month the Hgb can be kept within the target range with negligible oscillations. For the other patients the oscillations are even smaller.

**Table 3:** Patient 4: total EPO doses for different constant periods

Constant period	1 day	1 week	3 weeks	4 weeks
total dose (U)	55,598	56,964	56,721	57,695

## Conclusion

The presented NMPC algorithm to correct anemia in HD patients was tested in various in-silico experiments and showed excellent performance in stabilizing simulated Hgb levels. The introduced framework uses a system of non-linear hyperbolic PDEs, which previously has been adapted to individual patients using clinically measured Hgb levels to predict individual patient response to EPO treatment (Fuertinger et al. 2018). The proposed NMPC would allow to optimize anemia treatment based on single patient data sets only. It would further allow to set individual Hgb targets for certain patient groups as proposed by the KDIGO work group. Thus, the presented work is a first step towards fully individualized anemia therapy.

The conducted in-silico experiments show that a fixed optimization setting for the controller scheme is sufficient to correct the anemia of patients with very different characteristics in the underlying prediction model. However, the penalization of the control costs needs to be tuned on a subgroup or even per-patient level to minimize the amount of administered EPO. The NMPC method requires a comparatively long horizon to account for the system’s large time delay. We have determined the required horizon length experimentally. Given this horizon length, the controller can handle the delay of the system response to treatment and achieves to stabilize Hgb levels even when presented with simulated events such as bleedings, missed administrations and EPO dosing errors. The presented controller has been tested under the assumption that treatment with EPO can be provided continuously. While this is an interesting in-silico experiment, such a therapy is currently clinically not possible as there are no “EPO pumps”, similar to the insulin pumps used for diabetes treatment, available. As a first restriction on the control we have investigated to allow changes in the EPO rate less frequently resulting in constant EPO rates over several weeks. These lead to slight oscillations of the total RBC population around the target state. However, for periods of up to four weeks the oscillations are negligible.

Different controller schemes to correct anemia in HD patients have been proposed over the last years by various groups (Barbieri et al. 2015, 2016; Brier and Gaweda 2011; Brier et al. 2010; Martínez-Martínez et al. 2014; McAllister 2017; Nichols et al. 2011). Some of them have been successfully tested in clinical trials (Brier and Gaweda 2011; Brier et al. 2010) or even been implemented in the clinical routine (Barbieri et al. 2015, 2016). Most of the proposed solutions to correct anemia are based on MPC techniques. In general, the time-varying nature of the process, long time delays in the system, high inter-individual variability in the specifics or the response of the system to treatment and the need to react to unforeseen events such as bleedings and missed doses renders the problem of correcting and stabilizing anemia in HD patients more suitable to MPC based controller approaches than PID (proportional-integral-derivative) ones. Previously presented and clinically validated MPC approaches have been based on neural networks as the underlying prediction model (Barbieri et al. 2015, 2016; Brier and Gaweda 2011; Brier et al. 2010). Such models require large training and validation sets to find the optimal weights for the neural network. The presented approach, where the underlying model is a system of coupled PDEs, although one of the more complex ones currently proposed, allows to optimize anemia treatment based on single patient data sets only. The required data from a single patient is routinely measured in clinics (as presented in Fuertinger et al. 2018). Thus, the proposed NMPC approach would provide a fully personalized anemia treatment strategy that even allows the setting of individual Hgb goals as suggested by the KDIGO guidelines. Further, estimated model parameters and their longitudinal development in individual patients potentially allow to gain further insights in the specifics of renal anemia. It might allow to better understand why some patients do not respond to treatment (e.g. short red blood cell life span versus insufficient bone marrow reaction to treatment).

In summary, the presented NMPC algorithm has the potential to bring more patients in the Hgb target range while decreasing Hgb variability and EPO utilization. However, we are still two major steps away from clinically testing the proposed NMPC approach: First, the control structure needs to be changed such that EPO is only administered during dialysis treatments (in general three times per week). With the chosen approach we will be able to analyze the effect of reducing administration times on Hgb stability. Second, the patient-model mismatch and uncertainty in parameter estimates together with measurement noise need to be addressed. In order to deal with parameter uncertainty in the underlying model, for instance, so called “robust MPC” methods would need to be incorporated into the framework. In addition, model estimates will need to be updated on a regular basis using the measured Hgb data. This is currently under investigation by the group but is beyond the scope of this publication.

## A Appendix: Parameters for the model and the methods

In Tables 4, 5, 6, 7 and 8 we present all parameters and units utilized in our numerical experiments.

**Table 4:** Fixed model parameters, values and units

Par.	Meaning	Value	Unit
$\beta _1$	Proliferation rate for BFU-E cells	0.6	1/day
$\beta _2$	Proliferation rate for CFU-E cells	1.2	1/day
$\beta _3$	Proliferation rate for erythroblasts	0.723	1/day
${{\overline{x}}}_1$	Maximal maturity for BFU-E cells	3	Days
${{\underline{x}}}_2$	Minimal maturity for CFU-E cells	3	Days
${{\overline{x}}}_2$	Maximal maturity for CFU-E cells	8	Days
${{\underline{x}}}_3$	Minimal maturity for erythroblasts	8	Days
${{\overline{x}}}_3$	Maximal maturity for erythroblasts	13	Days
${{\underline{x}}}_4$	Minimal maturity for marrow reticulocytes	13	Days
${{\overline{x}}}_4$	Maximal maturity for marrow reticulocytes	15.5	Days
$\alpha _4$	Rate of ineffective erythropoiesis	0.09	1/day
$\alpha _5^0$	Intrinsic mortality rate for erythrocytes	0.002	1/day
${\widehat{\varOmega }}_5$	Age interval for erythrocytes, where	[14, 34]	Days
	neocytolysis is possible		
$\varepsilon$	Regularization parameter for the mortality rate	$10^{-8}$	–
$\tau _{E}$	EPO threshold for neocytolysis	80	mU/ml

**Table 5:** Fixed $\mu$-parameters, values and units

Parameter	Meaning	Value	Unit
$\mu _1$	Constant for the sigmoid apoptosis rate for CFU-E cells	0.5	1/day
$\mu _3$	Constant for the sigmoid apoptosis rate for CFU-E cells	0.5	Dimension-less
$\mu _4,\mu _5$	Constants for the sigmoid maturation velocity for marrow reticulocyte	2, 0.35	Dimension-less
$\mu _7$	Constant for the sigmoid maturation velocity for marrow reticulocytes	2.3	Dimension-less
$\mu _8$	Constant in the mortality rate for erythrocytes	$3.5\cdot 10^3$	$\mathrm {mU^{3}/ml^{2}}$
$\mu _9$	Constant in the mortality rate for erythrocytes	3	$\mathrm {mU^{3}/ml^{2}}$
$\mu _{10}$	Constant in the mortality rate for erythrocytes	0.1	$\mathrm {mU^{3}/ml^{2}}$

**Table 6:** Individualized model parameters and units

Parameter	Meaning	Unit
$c_\mathsf {tbv}$	Total blood volume	ml
${{\overline{x}}}_5$	Maximal life span for erythrocytes	Days
$S_{0}$	Rate at which cells are committing to the erythroid lineage	1/day
$E^\mathrm {end}$	Assumed constant endogenous EPO concentration in plasma	mU/ml
$T_{1/2}$	Half-life of Epoetin-$\alpha$	1/day

**Table 7:** Individualized $\mu$-parameters and units

Parameter	Meaning	Unit
$\mu _2$	Constant for the sigmoid apoptosis rate for CFU-E cells	ml/mU
$\mu _6$	Constant for the sigmoid maturation velocity for marrow reticulocytes	ml/mU

**Table 8:** Individualized parameters for considered patients

	Patient no.				
Parameter	1	2	3	4	5
$c_{{\mathsf {tbv}}}$	3567	7373	4543	6102	2600
$E^\mathrm {end}$	42.60	79.42	282.46	43	79
$\mu _2$	0.016	0.009	0.008	0.011	0.010
$\mu _6$	0.122	0.049	0.072	0.049	0.032
$T_{1/2}$	4.78	9.65	5.59	10.80	5.12
${{\overline{x}}}_5$	92.3	74.3	43.1	94.8	50.5
